# Cellular and Molecular Effects of SARS-CoV-2 Linking Lung Infection to the Brain

**DOI:** 10.3389/fimmu.2021.730088

**Published:** 2021-08-13

**Authors:** Linda Francistiová, Adrián Klepe, Géza Curley, Károly Gulya, András Dinnyés, Kata Filkor

**Affiliations:** ^1^BioTalentum Ltd, Gödöllő, Hungary; ^2^Department of Physiology and Animal Health, Institute of Physiology and Animal Health, Hungarian University of Agriculture and Life Sciences, Gödöllő, Hungary; ^3^Department of Cell Biology and Molecular Medicine, University of Szeged, Szeged, Hungary; ^4^Hungarian Centre of Excellence for Molecular Medicine - University of Szeged (HCEMM-USZ) StemCell Research Group, University of Szeged, Szeged, Hungary

**Keywords:** COVID-19, SARS-CoV-2, neuroinvasion, microglia, hypoxia

## Abstract

In December 2019, a new viral disease emerged and quickly spread all around the world. In March 2020, the COVID-19 outbreak was classified as a global pandemic and by June 2021, the number of infected people grew to over 170 million. Along with the patients’ mild-to-severe respiratory symptoms, reports on probable central nervous system (CNS) effects appeared shortly, raising concerns about the possible long-term detrimental effects on human cognition. It remains unresolved whether the neurological symptoms are caused directly by the SARS-CoV-2 infiltration in the brain, indirectly by secondary immune effects of a cytokine storm and antibody overproduction, or as a consequence of systemic hypoxia-mediated microglia activation. In severe COVID-19 cases with impaired lung capacity, hypoxia is an anticipated subsidiary event that can cause progressive and irreversible damage to neurons. To resolve this problem, intensive research is currently ongoing, which seeks to evaluate the SARS-CoV-2 virus’ neuroinvasive potential and the examination of the antibody and autoantibody generation upon infection, as well as the effects of prolonged systemic hypoxia on the CNS. In this review, we summarize the current research on the possible interplay of the SARS-CoV-2 effects on the lung, especially on alveolar macrophages and direct and indirect effects on the brain, with special emphasis on microglia, as a possible culprit of neurological manifestation during COVID-19.

## Introduction

Over the last twenty years, there have been two major new human Coronavirus outbreaks, namely the SARS-CoV in 2002 in China and the MERS-CoV in 2012 in Saudi Arabia (1, 2). The third novel Coronavirus outbreak occurred at the end of 2019 caused by SARS-CoV-2 in Wuhan, China (3, 4).

Based on their serological properties, members of the Coronaviridae genus can be grouped into α, β, γ and δ coronaviruses. The most pathogenic, pandemic-related human coronaviruses belong to the β group (5). Coronaviruses have a long, positive-sense, single-stranded RNA genome of 26 to 32 kilobases in size. The genome of SARS-CoV-2 contains 14 open reading frames that encode 27 proteins. At the 5’ region, 15 non-structural proteins required for viral replication are encoded. Open reading frames at the 3’ region encode those structural proteins - namely spike (S), nucleocapsid (N), an envelope protein (E) and membrane protein (M) - that are required for infection and induce host immune response (6, 7). In a genome comparison between SARS-CoV and SARS-CoV-2, it was found that 79% of both were identical, and the structural organization of their genomes was the same. In contrast to SARS-CoV, SARS-CoV-2 and MERS displayed less similarity, as only 50% of their genome was identical (8).

Although SARS-CoV, MERS-CoV, and SARS-CoV-2 have strong structural similarities and mainly cause lower respiratory tract infections and shortness of breath, these viruses have some unique features. A comparison of these three human pathogenic coronaviruses is listed in **Table 1**.

**Table 1:** Key comparison of geographical location and epidemiology data of SARS-CoV, MERS and SARS-CoV-2 caused infections.

	SARS-CoV	MERS	SARS-CoV-2	Reference
Outbreak	November, 2002	April, 2012	December, 2019	(3, 9, 10)
Location	Guangdong, China	Jeddah, Saudi-Arabia	Wuhan, China	
Transmission from animal to human	Coming from bats by infecting civets. Transmitted *via* close contact between humans	By consuming meat, or milk of infected camel. Only limited transition between humans	By touching, or eating of a not clearly verified animal, most probably pangolin. Transmitted between humans *via* close contact	(9–12)
Incubation time	2-7	5-6	2-14	(13)
Age	39.9 (1-91)	53 (36-66)	47 (all ages)	https://www.who.int/csr/sars/country/table2004_04_21/en/ https://www.who.int/csr/don/26-april-2016-mers-saudi-arabia/en/ https://globalhealth5050.org/the-sex-gender-and-covid-19-project/dataset/
Male:female ratio	1:1.13	2.03:1	1.22:1	
Mortality	9.6%	34.4%	2% (16^th^ june, 2021)	
Confirmed cases	8096	2519	177 419 783 (16^th^ june, 2021)	
Epidemic doubling time	4.6 to 14.2 days	90	6.4	(10, 13)
Predominant cellular receptor	ACE2	Dipeptidyl Peptidase 4 (DPP4, also known as CD26)	ACE2	(9, 14, 15)

The mildly pathogenic α Coronaviruses cause upper respiratory tract infections, while the highly pathogenic β Coronaviruses, including SARS-CoV, MERS-CoV, and SARS-CoV-2 cause serious lower respiratory tract symptoms (i.e., pneumonia), resulting in patients requiring respiratory support (16). Hence SARS-CoV-2 patients have a high risk of experiencing severe systemic hypoxia. Along with these, neurological symptoms may also develop, and the neuroinvasive tendencies of coronaviruses have been documented for almost all of the βCoVs, including SARS-CoV, MERS‐CoV (17), HCoV‐229E (18), HCoV‐OC43 (19) and the mouse hepatitis virus (20). SARS-CoV-2 also holds the potential for invading the nervous system. From the documented neurological symptoms, the mildest ones are anosmia and ageusia (sudden loss of smell taste) (21), but otolaryngeal symptoms, i.e., tinnitus, vertigo combined with a loss of hearing may appear (22). In severe cases, headache, seizures, delirium and even coma can develop (23). The presence of SARS-CoV-2 was confirmed in cerebrospinal fluid (CSF) taken from an encephalitis patient by next-generation sequencing (24) and by qRT-PCR indicating the presence of viral RNA in CSF (25, 26). In autopsy brain samples, Puelles and colleagues detected viral particles (27). Since the brain is one of the so-called immune-privileged sites of the human body, the investigation of anti-SARS-CoV-2 immunoglobulin G (IgG) production was also in focus, as the presence of antibodies in the CSF indicates intrathecal IgG production (28). However, in some cases, patients with the presence of SARS-CoV-2 IgG had normal CSF results, like ICP, cell counts, protein and glucose levels (29). In another, smaller trial, Barreras and her colleagues detected SARS-CoV-2 IgG in the CSF of patients with neurological symptoms. However, IgG levels did not correlate with the time between symptom development to sampling or disease severity (30).

**Figure 1:**
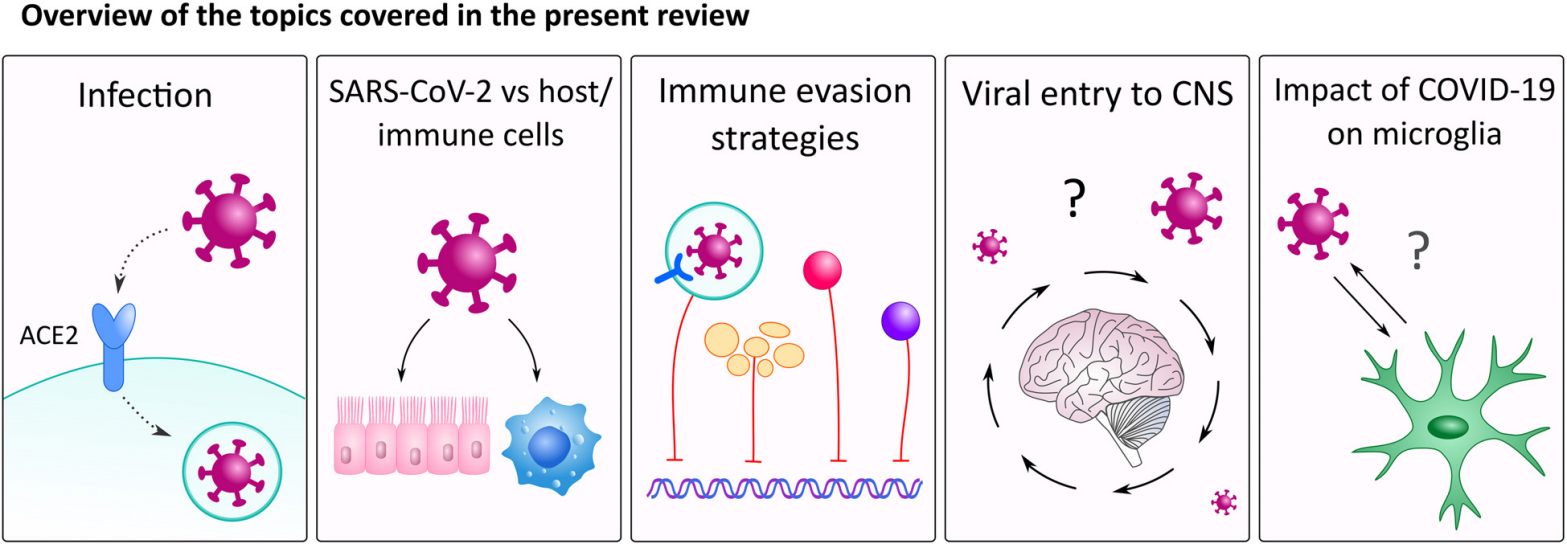
An overview of the topics covered in this review. Our review discusses how SARS-CoV-2 interacts with immune cells with a special focus on alveolar macrophages. Further, we will discuss how viral particles enter the brain and interacts with microglia cells.

There are several possible direct and indirect ways SARS-CoV-2 could interact with the CNS (31). In this review, we will discuss these possible interactions together with the impact of SARS-CoV-2 on alveolar macrophages and focus on the implications of hypoxia and hypoxia-induced factors on microglial cells (**Figure 1**).

## Interactions of SARS-CoV-2 With Host and Immune Cells

### Binding to ACE2 on the Host Cells

**Figure 2:**
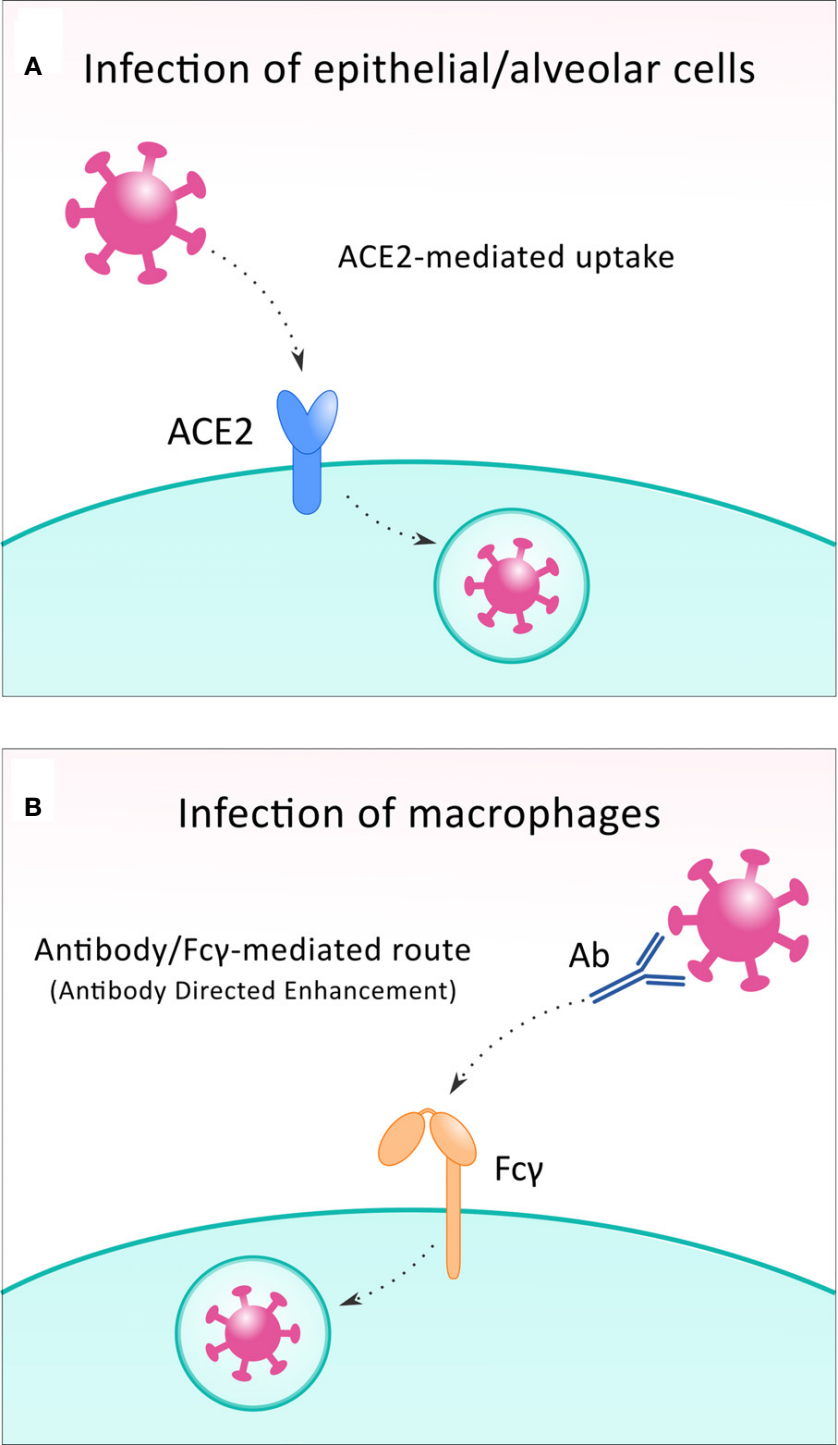
To infect the host cell, SARS-CoV-2 can exploit different receptors. In most infected cells, including epithelial and alveolar cells, the virus binds to ACE2 receptor **(A)**, while during infection of macrophages, Fcγ receptors are utilized in the antibody-directed enhancement mechanism **(B)**.

SARS-CoV-2 uses a transmembrane protein angiotensin-converting enzyme 2 (ACE2), a metallo-peptidase expressed not only in the respiratory epithelial cells but in almost every organ of the body. ACE2 is on the membrane of the target cells to establish infection (14). Based on its structure, the S-protein belongs to the class I fusion proteins. It is formed by two subunits, namely S1 on the N-terminal surface mediates receptor binding; and the S2 subunit, a transmembrane peptide on the C-terminal, is responsible for the internalization of the virion in the host cell (32).The binding affinity between the S protein of SARS-CoV-2 and the ACE2 is nearly ten-fold higher than in the case of SARS-CoV. Besides the attachment to the host ACE2 receptor, the priming of S protein by transmembrane protease serine 2 (TMPRSS2), a host membrane serine protease on the cell membrane, is also needed to permit the entry of the SARS-CoV-2 (33). Upon the formation of the bond, conformation changes take place in the S protein. It is cleaved by TMPRSS2, thereby allowing the release of the S2 subunit, and facilitating the entry of viral RNA into the cytoplasm of the host cell (32). As potential strategies for the treatment of COVID-19, the pharmacological inhibition of TMPRSS2 or the bond between human recombinant soluble ACE2 and the receptor-binding domain of S-protein could significantly reduce infection by SARS-CoV-2 (34) (**Figure 2A**).

The ACE2 receptor plays a physiological role in the renin-angiotensin system, and it indirectly affects the signaling pathway. ACE2 converts Angiotensin II (Ang II) into Angiotensin 1-7 [Ang-(1-7)], which binds to the Mas receptor that has a protective role in the lungs, as well as in other organs. However, in the case of Ang II binding to its receptor, vascular permeability can be facilitated by the JAK/STAT signaling pathway (35). During acute lung injury fibrosis, and when patients are exposed to the SARS-CoV-2 infection, ACE inhibitors and Ang II receptor blockers (ARBs) could moderate the lung injury by shifting the system towards the protective pathway [ACE2/Ang-(1-7)] (36). ACE inhibitors reduce hypertension which is common among patients with SARS-CoV-2. The concerns related to high blood pressure and COVID-19 are justified here because the co-occurrence of the two diseases is relatively high and this has been reported in several studies (37, 38). Furthermore, ACE2 is cleaved by increased activity of the transmembrane proteinase (ADAM17), indicated by the activation of the receptor of the tumor necrosis factor-α (TNF-α). Upon the release of cytokines such as IL-6, IL-1β and IFN-γ along with TNF-α due to SARS-CoV-2 infection, inhibition of the expression of ACE2 has been reported (35), leading to an imbalance in homeostasis and to inflammation and a cytokine storm (34).

### Primary Immune Evasion Strategy

The primary infected organ of SARS-CoV-2 is the lung causing acute respiratory distress syndrome (ARDS) and respiratory failure (39). Initially the virus infects the respiratory epithelial cells, which highly express ACE2 that provides an excellent entry for the virus (14). After having formed the bond between the S protein and ACE2 receptor, the virus can evade the immune system by effectively inhibiting the activation of TNF receptor-associated factors (TRAF) 3/6, which are key molecules in activating downstream signaling such as in interferon regulatory factor (IRF) 3/7 and nuclear factor kappa B (NF-κB) signaling pathways (14, 40). Deactivation of further transcription factors and the suppression of early pro-inflammatory responses through type I interferon (IFN-I) signaling, the coronavirus can start limiting antiviral response mechanisms (14, 41). The evasion of IFN-I-mediated innate immunity is likely orchestrated by the viral protein N which acts as an antagonist of IFN signaling. In this scenario, novel coronaviruses can counteract IFN expression by inhibiting the phosphorylation and nuclear translocation of transcription factors of the JAK/STAT signaling pathway (14, 39, 42). The suppressed expression of IFN-I can lead to an insufficient response of the host cells and inadequate clearance of viral infections (43). Thus, the activation of early antiviral programs appears to be prohibited contributing to the evasion of innate antiviral immunity by the coronavirus (14, 39). Through the suppressed expression of IFN-I, virions can regulate IFN-I signaling in infected macrophages, in which an increased level of pro-inflammatory cytokine expression (especially TNF-α and IL-6) *via* the NF-κB cascade is auto-amplified through positive feedback loops. These can contribute to triggering hyperinflammation and to developing a cytokine storm (14, 41).

**Figure 3:**
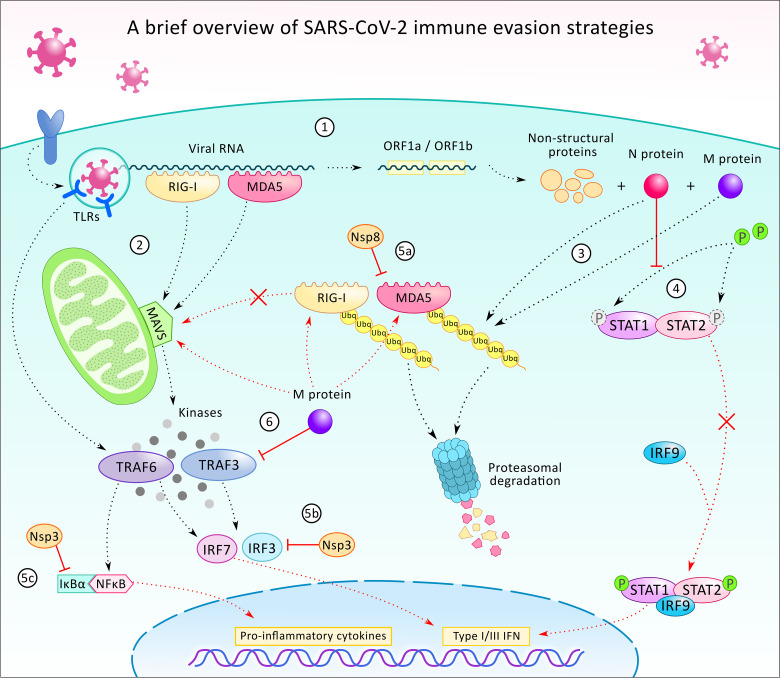
A brief overview of the SARS-CoV-2 virus’ immune evasion strategies. After infecting the host cell, the internalization of the viral particle, the transcription of the viral RNA starts with the translation of ORF1a and ORF2b, which generates proteins participating in the further transcription, host modulation, and immune evasion (1). In the cell, the presence of viral particles or viral RNA is being recognized by pattern recognition receptors such as toll-like receptors (TLR), retinoic acid-inducible gene I (RIG-1), and melanoma differentiation-associated protein 5 (MDA5) (2). TLRs (TLR 3, 7, and 8) are localized on endosomes and together with the cytoplasmatic viral sensors RIG-1 and MDA5, which orchestrate the translocation of NF-κB and IRF3 to the nucleus, leading to the expression of pro-inflammatory cytokines and type 1 and 3 interferons (INF-1/3). While TLRs activate TNF-associated factor 6 (TRAF6), RIG-1 and MDA5 act through mitochondrial antiviral protein (MAVS), to TRAF3/6, to IRF3/7. Viral protein N and M can alter the ubiquitination and degradation of RIG-1 and MDA5 sensors (3), thus restrains the activation of MAVS, TRAF3/6, and prevents IRF-3/7 and NF-κB signaling. N protein can also counteract IFN-1 production by inhibition of signal transducer and activator of transcription protein 1 and 2 (STAT1/2) phosphorylation and activation for further signaling (4). Additionally, some of the non-structural proteins (Nsp3, 8 and others - not shown) can also execute immunomodulatory functions, such as contributing to the inhibition of MDA5 (5a), IRF3 (5b), or stabilizing the NF-κB inhibitor IκBα (5c). Viral protein M also participates in immune evasion by blocking TRAF3 and binding to RIG-1, MDA5, and MAVS and preventing their interaction (6).

On the other hand, at a later stage, the activation of antiviral programs and the recruitment of non-infected immune cells occur. Virus particles released from the infected dead cells are recognized by endosomal RNA pattern recognition receptors (PRRs) which leads to the activation of the innate immune system and its cellular machinery (39, 44). Recent studies reported that not only the main RNA sensors, including cytoplasmic retinoic acid-inducible gene I (RIG-I) and melanoma differentiation-associated gene 5 (MDA5), have a key role in the detection and identification of coronavirus derived PAMPs (45, 46), but also pattern recognition toll-like receptors 3 and 7 (TLR-3 and TLR-7) are activated. TLRs induce IFN-I response and further increase the expression of IL-1β, IL-6 and TNF-α through MAVS, IRF3 and NF-κB cascades in immune cells (14, 44, 47). Therefore, the innate immune system is activated and macrophages are recruited to the lungs where hyperinflammation is triggered (14, 41) (**Figure 3**).

### Recruitment of Hypoxic Alveolar Macrophages

A less prevalent route for the immune evasion of SARS-CoV-2 is the infection of alveolar macrophages. While the “conventional” way of the entry of viral particles to the host cells is ACE2-mediated, in some cases, macrophages can be infected, and this happens *via* an antibody/Fcγ-mediated route. In this situation, SARS−CoV−2 virions are recognized by cross reactive neutralizing antibodies against seasonal coronaviruses (48); and then are taken up by the macrophage cells *via* Fcγ-receptors in a mechanism termed antibody directed enhancement (ADE) (49) (**Figure 2B**). Once taken up *via* Fcγ receptors, the virus particles inhibit the signaling of IFN-I in infected macrophages (14), leading to an increased expression of pro-inflammatory factors (IL-1β, IL-6 and TNF-α), which might result in hyperinflammation (14, 41). Besides this, the number of alveolar macrophages present during SARS-CoV-2 infection correlates well with disease severity (50). In the case of SARS-CoV, the delayed release of cytokines and chemokines was found in bronchial epithelial cells and macrophages at the early stage of infection *in vitro* cell experiments (51). Breathing difficulties caused by disrupted respiratory epithelial cells and the reduced alveolar partial pressure of oxygen (PO_2_) induce hypoxia, which causes inflammation and a hypoxic state in alveolar macrophages (52). The increased pulmonary expression of significant amounts of pro-inflammatory cytokines, NF-κB and hypoxia-inducible factor-1 α (HIF-1α) help uninfected, activated macrophages to invade into the alveoli, which recruit other immune cells such as CD8^+^ T effectors, and this produces more clones and causes tissue damage (50, 52, 53).

### Factors in Alveolar Macrophages During Hypoxia

In hypoxic alveolar macrophages, the expression of neurokinin-1 receptors is upregulated (52), and this hypoxic event induces the production of multiple factors such as reactive oxygen species (ROS) enzymes, transcriptional factors, and MAPKs (54). In the hypoxic state, when the O_2_ levels are at 10% instead of the normoxic 21%, the activated alveolar macrophages contribute to the release of H_2_O_2,_ leading to the inflammatory response (55). Likewise, in SARS-CoV-2, when alveolar macrophages are infected in the early phase of the disease, the secretion of pro-inflammatory cytokines (IL-1β, IL-6, IL-18 and TNF-α) and chemokines (CCL2, CCL3, CCL5) contributes to maintaining the emerged inflammation (14). Additionally, the autophagy of macrophages and activation *via* HIF-1α might result from hypoxia as well (56). HIF-1α, as a major transcription factor involved in response to reduced cellular oxygen levels, is significantly increased in the hypoxic state, while under physiological conditions, the levels of HIF-1α remain low (56). In response to hypoxia, increased mRNA, and protein levels of IL-8 and TNF-α have been observed, and these typical pro-inflammatory cytokines are released by macrophages and especially by microglia in the CNS (56).

Furthermore, the elevated expression of IFN-α/β and IL-6 can be detected (57), and secreted IFN-λ can disrupt the lung epithelial barrier by inhibiting the lung epithelial proliferation and its repair directly (58). While SARS-CoV-2 infects the macrophages, a viral cascade limits the antiviral response mechanisms by suppressing the activation of transcription factors (such as NF-κB and IRF3/7), thereby limiting the release of IFN-Is and allowing the secretion of the above-mentioned pro-inflammatory cytokines (14, 53). The positive feedback loops of inflammatory cascade by infected cells may contribute to hyperinflammation and the cytokine storm syndrome, the latter of which is also a key factor in extrapulmonary multiple-organ failure (51).

### Factors in the Blood Serum and Cells Infiltrated to the Bronchoalveolar Fluid

In the blood serum, high levels of C-reactive protein (CRP), ferritin, and D-dimer emerge during the course of COVID-19 infection (59, 60), suggesting an ongoing strong inflammation with self-perpetuating tendencies (61). Moreover, the increased serum levels of CCL7, CXCL10, and IL-1RA, and the presence of CCL2 and CCL7 in the bronchoalveolar fluid were found, pointing towards the activation of signaling towards macrophage recruitment. All of these changes might be associated with disease severity, lung disruption and have a potentially fatal outcome (62). To explain the elevated levels of these molecules, the high production of IL-6 and the activated macrophages should be taken into account. IL-6 is a cytokine of pleiotropic activity, but during viral infections, IL-6 is considered one of the most important cytokines for its regulation of T-cell response, prevention of viral-induced apoptosis of lung epithelial cells, regulation of IgG isotype switching, and other functions (63, 64). Moreover, an increased IL-6 level in the serum is associated with a negative prognosis in patients with SARS-CoV-2 (65). In COVID-19 patients, the gene expression of subsets of CD4^+^ and CD8^+^ T cells were observed in the bronchoalveolar fluid, and these subsets were activated differently; moreover, the B cell populations were altered, while the monocyte infiltration was minimal (53, 60). Furthermore, IgG levels declined about two months after symptoms of the early onset of the disease, but the recovered patients maintained high spike-specific IgG titers (60). The findings show that many factors and cells can be present in the blood and in the tissue fluid after a lung infection.

### T Cells and Autoantibodies Involvement in the CNS

A key component of any immune response, including the response to SARS-CoV-2, is the activation of CD4^+^ and CD8^+^ T cells and the subsequent production of neutralizing antibodies (66). It is very interesting that the profile of anti-SARS-CoV-2 antibodies in the plasma and CSF differs within the same COVID-19 patient, suggesting a compartmentalized immune response within the brain (67). Such divergent humoral response indirectly supports neuroinvasion of SARS-CoV-2 during acute infection. Longitudinal profiling studies of neutralizing antibodies against SARS-CoV in 2005 revealed that recovered SARS patients had developed high levels of neutralizing antibodies, while patients with a shorter illness duration displayed higher neutralizing antibody activity compared to patients with a longer illness duration (68, 69). This suggests that antibody responses do indeed play a role in determining the disease outcome. Based on the similarities between SARS-CoV and SARS CoV -2, it is likely that neutralizing processes against SARS-CoV-2 antibodies also develop; and this could contribute to the course, duration, and possible termination of acute infection in the lung and other organs, including the CNS.

As is well known, the CNS contains CD4^+^ and CD8^+^ T cells, which patrol and protect the borders of CNS, while CD8^+^ T cells provide a cytotoxic defense against viral infections in the brain parenchyma (70–72). Aside from the direct cytotoxic reactions in the presence of viral particles in the brain, local cytokine and chemokine milieu is important for T cell generation, retention and infiltration to the brain. Thus, T cells might contribute to cytokine production and further exacerbate inflammatory conditions in the brain by paracrine signaling to microglia (73). For this reason, it might be worthwhile examining the T cell involvement during the course of SARS-CoV-2 infection in the brain-resident T cells. During CNS infection with neurotropic viruses, T cells infiltration is followed by B cells infiltration to ensure local secretion of antibodies since a passage of antibodies from serum into the brain is prevented by BBB (74).

Recently, several reports emerged describing the presence of autoantibodies in high proportions of patients with the most severe cases. These reports show that about 10% of the tested patients with severe COVID-19 had antibodies against the type I interferon molecules and other autoantibodies against proteins of blood vessels, heart, and brain (75, 76). These findings could explain the delay in the onset of the severe COVID-19 symptoms, how the “long COVID” develops, and why sometimes lung damage continues to progress long after the virus is no longer detectable in the body. Intensive research on this issue is currently ongoing. It could provide clues on the possibility that some people might be predisposed to producing autoantibodies and hence potentially be more likely to develop severe COVID-19 disease.

## Entering the Brain by SARS-CoV-2

**Figure 4:**
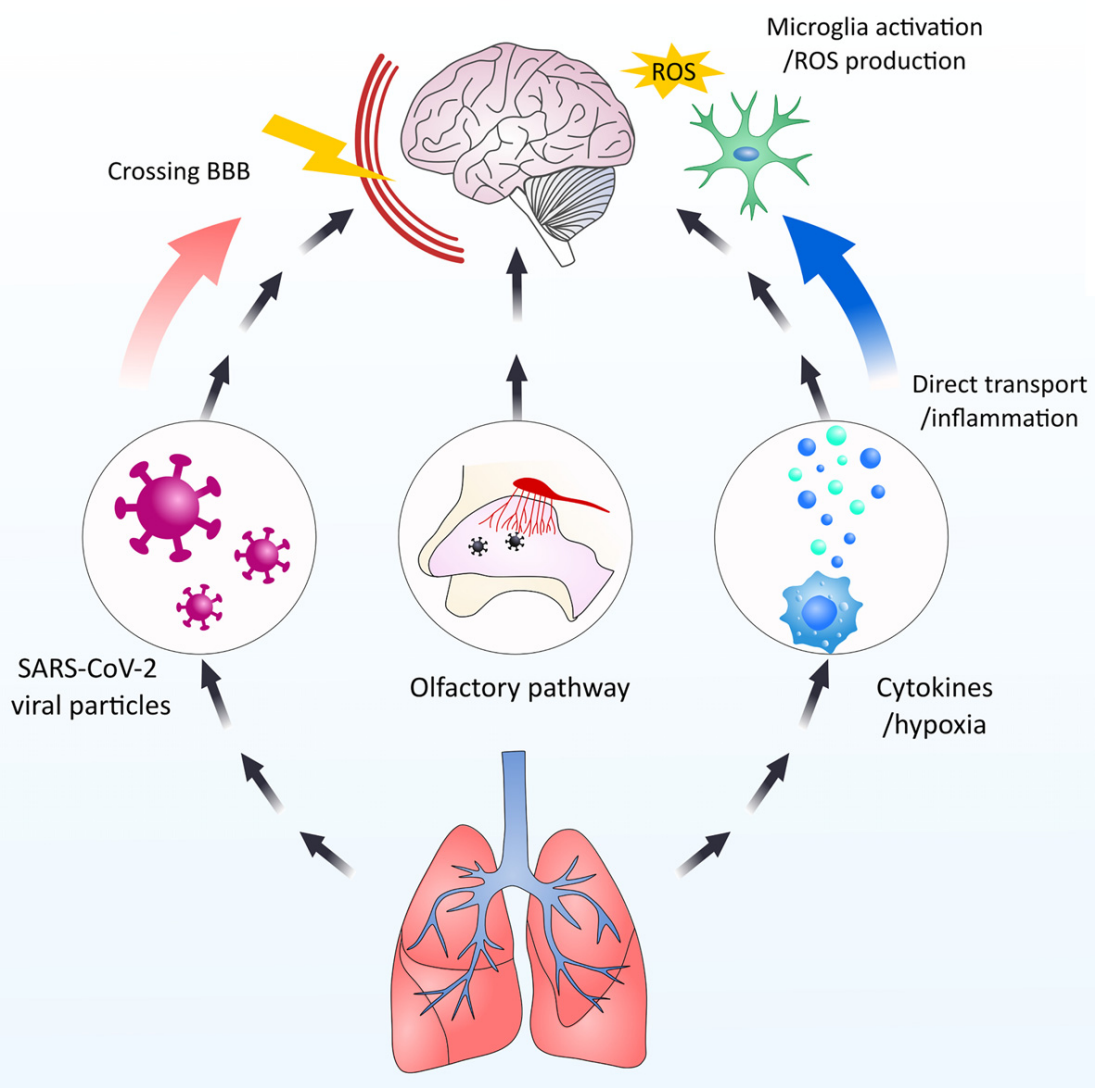
Schematic overview of the possible interactions of SARS-CoV-2 and CNS. SARS-CoV-2 particles could potentially and negatively influence the CNS by multiple means. 1. Directly entering the CNS *via* crossing the BBB. 2. By-passing the BBB by entering *via* the olfactory nerve. 3. Indirect influence *via* microglia activation by cytokines infiltration from the periphery and hypoxia-induced activation.

COVID-19 patients may be at a higher risk of infection of the nervous system, encephalitis and developing cognitive decline after displaying symptoms such as olfactory and gustatory dysfunctions, among others (72). Although CNS is not the primary organ affected by SARS-CoV-2, viruses can directly invade the CNS and damage nerves and neurons that were identified as targets of this viral infection (31). SARS-CoV-2 can migrate by retrograde or anterograde neuronal transport through motor proteins by infecting motor or sensory nerve endings (31). Moreover, SARS-CoV-2 can gain access to the brain either through the olfactory tract or *via* the lung epithelium (77) (**Figure 4**). In several COVID−19 cases, neurological complications were reported, including mild symptoms such as confusion, fatigue, anxiety, reversal of sleep-wake cycle, and headaches (78). But also severe neurological syndromes such as encephalopathies (79) (often with delirium or psychosis), meningo-encephalitis (25), ischemic stroke (80), acute necrotizing encephalopathy (81), and Guillain-Barré syndrome (82). An impressive cohort study by Peterson et al. provides a comparative analysis of neurological data from patients with COVID-19-related neurological disorders. Interestingly, many of the patients had only mild respiratory symptoms, showing that these complications were not related to the severity of the respiratory COVID−19, and thus suggesting that the primary affected organ was the brain. However, none of the patients tested for the presence of SARS−CoV−2 protein in CSF were positive (83). Another cohort study focused on neurological complications of COVID−19 additionally showed that the primary neurological impairments in younger patients comprise psychiatric diagnoses with alterations in mental status and encephalopathy or encephalitis, while in older patients (>60 years), the leading neurological complications were of cerebrovascular origin (84). Although extremely valuable, these studies present data sets collected from a small number of patients, and as authors remarked, it could show a bias towards severe disease. At the same time, they highlight the importance of future studies of the neurological complications accompanying COVID-19 and follow-up studies of the affected patients that will help uncover the long-term effects of the disease. In this section, we will explore the possibilities of the SARS−CoV−2 to directly infect the brain, while in the later sections, we will discuss the indirect, possibly immune-overactivation-based routes of COVID−19-related CNS damage.

### Viral Entry Through the BBB

The CNS, sometimes described as “immune privileged”, is under normal conditions carefully sheltered against the invasion of pathogens from the outside environments as well as from the periphery. At the forefront of the brain’s immune privilege is the neurovascular unit, which consists of endothelial cells, associated blood-brain barrier (BBB) tight junctions (TJ), basal lamina, pericytes, parenchymal cells, astrocytes, neurons, and interneurons. The neurovascular unit ensures the maintenance of the BBB, cerebral homeostasis, and the cerebral blood flow (85). To further ensure protection against unwanted inflammation, along with the BBB, which separates the brain parenchyma from the peripheral blood (86), other biological barriers are present in the brain. The blood-retina barrier (BRB) guards the interface with retinae (87), and the blood-cerebrospinal fluid barrier (BCSFB) isolates the brain from contact with the CSF (88). Despite such sophisticated defense mechanisms, the invasion of the CNS by pathogens can occur. The entry of various pathogens, including viruses, into the CNS can be facilitated *via* multiple different routes: such as transcellular (e.g. by receptor-mediated transport of the viral particle or by pinocytosis uptake without disruption of the cellular barriers), paracellular (e.g. by disrupting the tight-junctions of the BBB and increasing the permeability of the BBB), as intracellular cargo (the “Trojan horse” method when the viral particle present inside of a cell is allowed to enter the CNS by circumventing the BBB) (89). Transcellular entry occurs as a receptor-mediated uptake or pinocytosis, but the cellular barriers are not disrupted. A potential target of SARS-CoV-2 infection is the endothelium, especially brain microvascular endothelial cells (BMVECs), which form the BBB, among other cell types. After crossing the basement membrane of the lung epithelium, the virus reaches the blood vessel and then the BMVECs, to be transported *via* the cellular pathway by binding to ACE2 receptor or *via* an intercellular pathway across the BBB into the brain (90). Potential direct viral infection can occur by the biding of the SARS-CoV-2 to ACE2 receptor on the surface of BMVECs. Moreover, internalization can lead to the production of ROS and the increased secretion of proinflammatory cytokines as well as chemokines (i.e., CXCL10) that can be detected in serum (91). And a recent study suggested that the coronavirus S1 protein might be cleaved from the virus by the host cell’s proteases and subsequently be available as a single infectious unit for crossing the BBB. The authors mention that this process could be executed *via* a mechanism similar to adsorptive transcytosis and uptake by peripheral tissue and be ACE2-independent. Once the protein enters the brain parenchyma, it might remain biologically highly active and toxic, and thus induce detrimental responses in the brain without the whole virions being involved (92). It should be added that this research was performed on mouse models and this mechanism could not be reproduced on a human induced pluripotent stem cell (iPSC)-based BBB *in vitro* model. Nevertheless, this result suggests an interesting avenue that deserves investigation.

Both inflammation and a potential cytokine storm induce the loosening of the TJ complex with alterations in the expression of TJ proteins such as in ZO-1, occludin, claudin-5 and VE-cadherin (93). Furthermore, cytoskeletal remodeling without a tight BBB leads to both vascular leakage and coagulation (57). The viral infection itself and inflammatory molecules bring about an enhanced permeability of the BBB (91). Through the compromised BBB, blood monocytes can infiltrate the CNS and produce a direct infection followed by the rapid activation of microglia among other glial cells (94). The balance- and type of cytokines and their cumulative effects at the BBB are complex and regulated by multiple signaling pathways and cell types. Next, the whole cascade results in a leaky and impaired BBB. Perrin and colleagues reported COVID-19 cases in which an elevated astroglia marker, S100B level was detected as a sign of increased BBB permeability (95). When an intense systemic inflammatory response occurs, more virus and peripheral cytokines such as IL-1β, IL-6, IL-17 and TNF-α, among others, can enter the brain *via* the damaged BBB. Hence, exacerbated, or triggered neuroinflammation by activating microglia, as the key cellular mediators of this process, may be developed and intensified by Th17 cells transmigration to the brain parenchyma and IL-17 upregulation, both caused by the augmentation of TNF-α (96). Together with the SARS-CoV-2 infection, the cascade promotes a cytokine storm that results in the increased secretion of pro-inflammatory factors (such as MIP1-α, IP-10, G-CSF, CRP and ferritin, among others) (97). Furthermore, the bond between cytokines/chemokines and their specific receptors on the cerebral microvascular endothelium provokes in addition to neuroinflammation, BBB breakdown and encephalitis (97).

### Viral Invasion Through the Choroid Plexus

The choroid plexus is a single layer of epithelial cells located in the brain’s ventricular system, and it produces cerebrospinal fluid. Neighboring cells of the choroid plexus are interconnected *via* TJs and form the BCSFB and contribute to the homeostatic regulation of the microenvironment in the brain (98). Recently, it has been found that certain cells of the choroid plexus express the ACE2 and other SARS-CoV-2 entry factors such as the TMPRSS2 (99). The same study using iPSC-derived brain organoids demonstrated that the infection of the choroid plexus cells by the SARS-CoV-2 causes substantial damage to the epithelium and a subsequent leakage across this important barrier. At the same time, the group did not observe infection of neuronal cells. These results overall suggest that the neurological symptoms observed in COVID-19 patients might be a secondary consequence of the infection of supportive CNS cells rather than the direct infection of neuronal cells (99). This hypothesis has yet to be confirmed experimentally, although it seems to be in line with the findings of a post-mortem study of the brains of 43 COVID-19 patients. The authors found no evidence of CNS damage caused directly by SARS-CoV-2 but found strong microglia and astrocyte activation and infiltration of cytotoxic T-lymphocytes (100), which might be a consequence of the disrupted brain barrier. On the other hand, a new study revealed the selective susceptibility of dopaminergic cells to SARS-CoV-2 infection and observed inflammatory and senescence on the transcriptional level (101), indicating that this issue among COVID-19 patients might require special attention.

### Olfactory Pathway for Invasion of the CNS

Although the main route for the most viral infections of the lower airways is considered to be the oral-lung aspiration axis (102, 103), research of the novel coronavirus SARS-CoV-2 suggests that in this case, the more prevalent entry is *via* the aspiration of oropharyngeal mucus originating from the nasal cavity and containing the viral inoculum, into the deep lung. Evidence supporting this hypothesis comes from the knowledge that the nasal lining has a higher concentration of the ACE2 receptor compared to the lower airways, creating a gradient of high susceptibility of infection in the nasal cavity to lower parts in the deep areas of the lung (104). Moreover, the autopsies of patients who died as a consequence of COVID-19 showed that the macroscopic appearance of the infected areas of the lungs was described as “patchy, segmental and peripheral” (104). If the nasal surface indeed serves as the dominant initial site for SARS-CoV-2 infection, the threat of spreading into the olfactory nerves should be considered. The olfactory nerve projects from the nasal cavity directly into the olfactory bulb of the brain, while in the mucosal part of the nasal cavity, ciliated dendrites of the nerve extend to the mucus-lined airway space. There they gather odorant information and transmit it *via* olfactory sensory neurons’ axons through the cribriform plate directly to the brain (105). In this way, the olfactory sensory neurons are constantly exposed to the environment – including the potentially present pathogens (106); and hence serve as a direct single-cell route for neuroinvasion. Interestingly, the same study showed that despite the fact, that viruses can sometimes enter the CNS *via* the olfactory nerves and successfully enter the olfactory bulb of the brain while by-passing the protective barriers, another safety check is probably in operation in the olfactory bulb. In fact, after the infection of the olfactory bulb *via* the olfactory nerve, the infection was halted before it had the chance to spread to different CNS areas (106). To achieve such an inhibition of the spread of viral infection, the immune system needs to be ready to react in a way that neutralizes the viral particle yet does not damage the neurons. Such noncytolytic clearance is commonly facilitated by IFNs. INFs are cytokines primarily produced by lymphocytes (CD8^+,^ CD4^+^ and natural killer cells (NK)) and serve as the primary protective measure against viruses (107). In the study of Moseman et al. (106), after the neuroinvasion of the viral particles to the brain, the first line of defense was represented by microglia, which shortly after the infection showed evidence of activation. The activation of cells characterized by the up-regulation of antigen-presenting molecules MHC I, CD80 and CD86, enabled microglia to present viral antigens to infiltrating CD8^+^ T cells. CD8^+^ T cells subsequently exert antiviral pressure and stop the viral spread throughout the CNS. It is interesting that in this study, microglia were not infected by the virus in question (vesicular stomatitis virus), yet it was able to present the viral antigens to T cells. This means that microglia acquired the antigen from nearby neurons and cross-presented them to the T cells (106). The scenario of the olfactory nerve infection by SARS-CoV-2 seems to be plausible as it has been reported that in many cases, the patients developed progressive ageusia and anosmia (108). Interestingly, another recent study presented evidence of the olfactory transmucosal invasion of SARS-CoV-2 infected individuals’ brains. These findings are supported by the detection of SARS-CoV-2 RNA as well as viral protein in the brain regions. Moreover, the authors described morphological changes of the tissue associated with such an infection, collectively supporting the SARS-CoV-2 neurotropism and the viral spread along the neuroanatomical structures receiving projections from the olfactory tract (109).

To determine how exactly the SARS-CoV-2 virus enters the olfactory nerve and propagates through it into the CNS, the ACE2 expression levels in the olfactory nerve and the CNS need to be investigated. Regardless of the manner of entry, once the virus successfully infects the CNS *via* the olfactory nerve, further regions of the brain are involved, such as the piriform and infralimbic cortices, basal ganglia, and dorsal raphae nuclei, while other areas such as the thalamus and hypothalamus are less frequently reported as positive (110). Such a distribution pattern would suggest a transneuronal spread (111). Despite these findings, the clear-cut neuro-invasive potential of SARS-CoV-2 is still yet to be determined, as some research also indicates that only epithelial (sustentacular) cells of the nasal mucosa can be infected, not olfactory neurons (112).

## The Impact of SARS-CoV-2 on Microglia

### Gene Expression Modifications *via* HIF-1α

**Figure 5:**
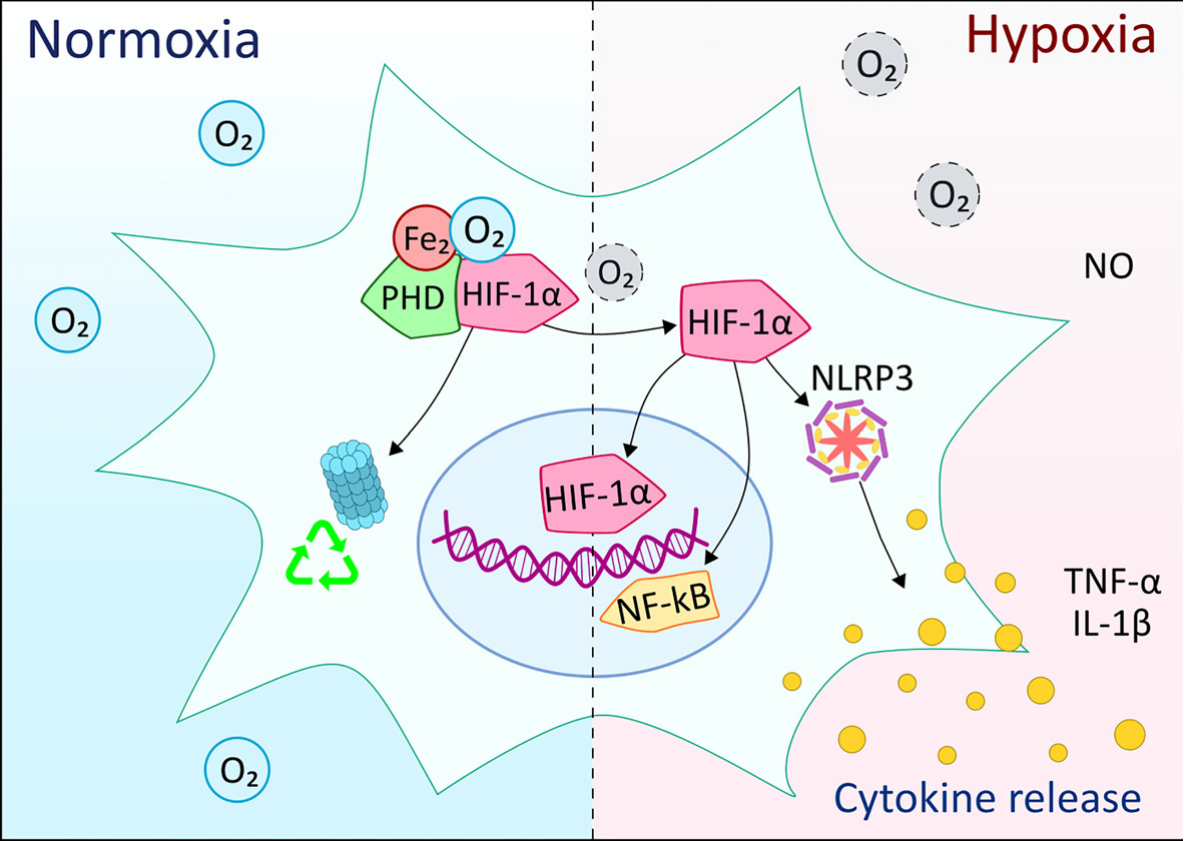
Overview of the effects of hypoxia on microglia cells compared to normal oxygen levels. HIF-1α is one of the main components of the response to hypoxia in myeloid cells. Under normoxic conditions, the oxygen molecules participate in the HIF-1α hydroxylation, ubiquitination and subsequent degradation by the proteasome complex. In hypoxia, the lack of oxygen molecules prevents ubiquitination and degradation of HIF-1α, which can then translocate to the nucleus and, together with other co-activating elements, initiate the expression of pro-inflammatory molecules. At the same time, HIF-1α activation might involve crosstalk with NF-κB, NLRP3 inflammasome, as well as nitric oxide (NO) production along with pro-inflammatory cytokines.

The family of HIF proteins is transcription regulators that respond to the prevalent oxygen levels and modify the gene transcription rates of specific DNA sequences. HIF-1 is a dimer that consists of HIF-1α and HIF-1β subunits. While HIF-1α is present at very low levels during normoxia, HIF-1β is continuously transcribed. In normoxic conditions, HIF-1α is hydroxylated in the presence of iron, oxygen, and 2-oxoglutarate, then HIF-1α undergoes ubiquitination and is destroyed (**Figure 5**) (113). However, in hypoxemic conditions, the oxygen required for HIF-1α ubiquitination is lacking. Therefore, HIF-1α does not degrade, but translocates to the nucleus, where it binds with HIF-1β, then recruits coactivator proteins at the hypoxia response element. Numerous target genes that assist in hypoxic adaptation are upregulated, such as VEGF, which induces angiogenesis, or erythropoietin, which generates more erythrocytes (114). However, some genes are downregulated (e.g., PDK1), and this reduces the oxygen consumption of the mitochondria. The regulation of genes that boost glucose flux to pyruvate causes a switch from oxidative to glycolytic cellular metabolism (115). Furthermore, through endothelial nitric oxide synthase modulation, HIF-1α can also affect pulmonary circulation (116).

The interaction between HIF-1α and glucose levels is particularly important, as glycolytic flux is required for SARS-CoV-2 replication since high glucose levels promote viral replication and pro-inflammatory cytokine expression (117). Moreover, ROS are potent inducers of HIF-1α (118). The mitochondrial ROS (mtROS) production caused by SARS-CoV-2 stabilizes HIF-1α, which then upregulates IL-1β expression as well as glycolytic genes. These findings explain why uncontrolled diabetes can lead to lung dysfunction and a maladaptive immune response in patients with severe COVID-19 symptoms. They also suggest that the mtROS/HIF-1α/glycolysis axis could be a target for treating this disease (117).

### Hypoxic State and HIF-1α Expression

Hypoxia plays a central role in endothelial activation and inflammation, and it creates a positive feedback loop *via* the reduction of the expression of the complement regulator CD55 by HIF-1α, IL-2, and TNF-α (119). In hypoxic and inflammatory microenvironments, HIF-1α is a key driver of myeloid cell response: (I) It modifies cellular energetics, (II) it upregulates glycolytic enzymes and glucose transporters to allow ATP generation under hypoxic conditions, and (III) it prevents the apoptosis of innate immune cells. In chronic infections, however, HIF-1α prevented excessive lymphocyte recruitment into lung interstitium and pathological immune consequences of the host (120). During the infection of SARS-CoV-2, the infected monocytes express higher levels of pro-inflammatory cytokines and multiple forms of IFN, such as IFN-α, β and γ (121). The presence of TNF-α, IL-1β, IL-6, and HIF-1α are associated with the COVID-19 cytokine storm. Dysregulated blood glucose levels in diabetic patients are a substantial risk factor for the severity of the disease. At the same time, elevated glucose levels might further augment TNF-α, IL-6, and IFN-α/β expression and initiate a vicious cycle of immune hyperactivity (121).

### Hypoxic and Normoxic Conditions

In patients with COVID-19, pneumonia and vascular permeability are related to the elevated thrombosis (119), which is stimulated by a hypoxic state in which the hypercoagulability is increased along with HIF-1α expression (122). Cells adapt to it by activating HIF-1 and HIF-2, which promote the expression of a wide array of genes involved in cell survival and specifically endothelial cell adaptation and energy metabolism. A transition from HIF-1 to HIF-2 suggests an adaptation from acute to prolonged hypoxia, although most genes can be regulated by both (123). HIF-1α has a short cytosolic half-life in normoxic conditions, low basal levels, and a high turnover, while in hypoxemic conditions, it is conserved (113). The cytosolic accumulation of succinate also prevents HIF-1α breakdown (124).

Besides being a coactivator of HIF-1α, pyruvate kinase isozyme 2 (PKM2) plays a key role in glycolysis and mediates acute inflammation (125). PKM2 dimers can directly interact with HIF-1α *via* nuclear translocation (125, 126). In the nucleus, HIF-1α regulates the adaptor response to hypoxia (125), transcribes pro-inflammatory cytokines and glycolytic machinery (124), especially IL- 1β, a key factor involved in acute and chronic inflammation and generation of fever response, and IL-6. This pro-inflammatory shift is accompanied by the downregulation of anti-inflammatory cytokines such as IL-10 (126). This condition is distinguished by excessive neutrophil-predominant inflammation and by disrupting the alveolar-capillary barrier that causes acute hypoxemic respiratory failure (127, 128).

The HIF-1 signaling pathway can interact with ErbB, PI3K-Akt, mTOR pathways and their connected proteins (129). *Via* a cascade: PI3K activation phosphorylates Akt, which activates mTOR. It is followed by the activation of 4E-BP1 and the eIF4 complex by Akt and mTORC1, which causes a translation of HIF-1α, an effector protein that initiates transcription and translation of host-specific genes (129, 130). The TNF signaling pathway is interlinked with HIF-1 signaling; hence, it can also increase HIF-1α by activating PI3K-Akt, MAPK, and NF-κB pathways and lead to the overexpression of HIF-1α mRNA translation and protein synthesis, but not affect its stability (131).

### Microglia in Health and Disease

Microglia are the resident innate immune system cells in the brain and the first responders to damage of the CNS (132–135). Besides the maintenance of neuronal homeostasis and participating in the formation of the BBB (136), microglia perform critical physiological functions such as synaptic pruning (137), trophic support of the neurons, the removal of apoptotic debris (138), and continuous immune surveillance (139–141). Whenever the brain’s homeostasis is compromised, microglia can rapidly adapt their phenotype and functions in response to the signals from their environment, such as cellular damage or the infiltration of foreign entities (142). Depending on the nature of the activating signal, microglia change their phenotype into the phagocytic or cytokine-producing state. Often, this duality of activation states is referred to as M1 (IFN-γ-dependent classical activation; pro-inflammatory activation) and M2 (IL-4-dependent alternative activation; repair-inducing/phagocytic) activation states (143), during which microglia undergo morphological and metabolic changes in order to efficiently remove the detected threat (143). Although the long-standing notion of microglial cells having M1/M2 activation states is widely accepted (144), a growing body of research indicates that this model might be oversimplistic considering the complexity of microglial effector roles in both health and disease (145). However, as the M1/M2 model is in use within the context of microglial responses to the presence of pathogens, we will adhere to this nomenclature in the review.

At the time of homeostasis, the quiescent state of microglia is maintained mainly *via* the absence of activating factors, and it is supported by pacifying signals originating from neuronal and astroglial cells (e.g. CX3CL1 and CD200) (146). However, in the presence of an activating signal (e.g. a recognized bacterial or viral particle), microglia rapidly switch from the homeostatic stage with ramified morphology to the classically activated (M1) form. An outcome of an M1 polarizing event is the production of M1-associated factors such as pro-inflammatory cytokines: TNF-α, IL-1α, IL-1β, IL-6, IL-12, IL-23; chemokines; REDOX molecules and other co-stimulatory proteins while phagocytosis is inhibited during M1 activation (144). However, in the case of detection of cellular damage, sterile injury or neurodegenerative disease, M2 activation occurs, and the activation pattern includes events that lead to inflammation resolution through anti-inflammatory factors (TGF-β, IL-10, IL-13, VEGF, EGF and Arg1) in an attempt to re-establish homeostasis (147). One of the results of M2 activation is the transformation of the cells into the amoeboid type and the activation of phagocytosis, which attempts to remove cellular debris and recover tissue equilibrium (143).

### Inflammation in Viral Infection

As an innate immune response for viral infection in the CNS, microglial cells transit towards a proinflammatory M1 state and rapidly proliferate, leading to enhanced phagocytic activity of microglia (148). During the rapid division of microglial cells, the so-called Warburg effect occurs, in which the ATP is rapidly generated by the enhanced glycolytic energy pathway. This metabolic adaptation is an essential component for the polarization of microglia besides the increased production of cytokines such as IL-6 and TNF-α (148). When microglia polarizing to the M1 state during viral infection, the cells are likely to be exposed to hypoxic environments, which also activates HIF-1α expression (148). IFN-α/β genes play a pivotal role in the potent immune response against viral infections (149). Due to the interaction between the INF-α/β signaling and the IFN-γ pathway, microglia may become more responsive to the virus by its INF-γ antiviral program (150). Furthermore, the results of Zhou et al. clearly show that IFN priming prior to tissue cell infection with SARS-CoV results in the augmented expression of several molecules involved in the induction and upregulation of signaling pathways of IFN-β, among other factors (151).

Although astrocytes are the main source of IFN-α/β in mice (152), in the case of direct viral encephalitis, infected microglia also contribute to IFN-I production, which is dependent on signaling *via* the IFN-α/β receptor in an MDA-5 dependent pathway (153). Besides the IFN family, the critical role of IL-10 was found during an emerging inflammation caused by a viral infection. CD4^+^ cells are the pivotal source of IL-10, which cytokine blocks the production of IFN-γ of CD4^+^ lymphocytes. In the absence of IL-10, higher levels of INF-γ mRNA and protein were produced by CD4^+^ cells without alterations in the functions of CD8^+^ lymphocytes. In coronavirus infections, not only high numbers of microglia expressed iNOS and MHC-II, but the viral clearance was also more rapid in lesions of IL-10 deficient mice (154). Overall, in viral infections, the absence of IL-10 results in demyelination and cell death, while the presence of IL-10 protects against tissue damage.

Along with the above-mentioned processes, the inflammatory stimulus in COVID-19 can also be initiated by the cascade of a viral infection that can, in turn, activate the lung macrophages and affect the neutrophil influx; and this indirectly leads to NLRP3 inflammasome activation (155). NLRP3 inflammasome is crucial for the induction of acute lung injury when IL-1β signaling is induced by hypoxia. Similar to hypoxic alveolar macrophages in the lungs, microglia in the brain are the primary source of IL-1β (152). A higher level of another cytokine, IL-6, was observed in parallel with the downregulation of microglial functions in the case of hypoxia. Here, the pathological driver is the existent IL-6-mediated cytokine storm, which appears within two days, leading to neurotoxicity in less than a week (156). While astrocytes are the source of IL-6, an increased hypothalamic IL-6 level was observed along with microglia activation caused by lung infection (157). In contrast, a blockade of microglia in a hypoxic state led to induced astrocytic IL-6 production. A specific therapeutic blockade of circulating IL-6 can be achieved using tocilizumab or siltuximab, but the efficiency of such treatment is relatively low (158).

### The Role of Self Molecules in Microglia Activation

In general, when cells are injured, the normally intracellular molecules, such as ATP, appear in the extracellular space and serve as a danger-associated molecule pattern (DAMP), which is recognized by the PRRs of the macrophages (157). Therefore, this inflammatory cascade can be initiated by the activation of macrophages by upregulated cytosolic PRR signaling pathways in response to the tissue damage caused by SARS-CoV-2. Through the blood circulation, having released into the brain parenchyma, DAMPs and SARS-CoV-2-derived PAMPs can reactivate microglia. The activation of PRRs on microglia initiates the antiviral cascade *via* e.g.: the NF-κB (158). The production of IFN-I and -III promotes intracellular antiviral defense and the production and release of microglia and macrophage-dependent IL-1β and IL-6, are the primary response to viral infection (157). However, like M protein or N protein in the SARS-CoV, in SARS-CoV-2 these viral proteins can block the formation of the TRAF3, TANK and TBK1/IKK complex (159); and thereby inhibit the production of IFN-I, which happens in the case of dendritic cells (160). Additionally, elevated levels of IL-12, p40, TNF-α, IL-15, IL-6, and IL-1β, on both mRNA and protein levels, were observed in a neuron culture infected by a murine neurotrophic β Coronavirus then both in non-neurotrophic virus, MHV-2 infected cells and in the uninfected control cultures (161). Overall, microglia may contribute to viral control and nervous tissue damage in response to SARS-CoV-2 CNS infection (158, 159, 162); and thereby inhibit the production of IFN-I, which happens in the case of dendritic cells (160). Additionally, elevated levels of IL-12, p40, TNF-α, IL-15, IL-6, and IL-1β, on both mRNA and protein levels, were observed in a neuron culture infected by a murine neurotrophic β Coronavirus then both in non-neurotrophic virus, MHV-2 infected cells and in the uninfected control cultures (161). Supporting the idea, the quantities of cytokines are positively correlated with the viral titer, and the cytokine levels are high in the absence of microglia (153).

## Conclusion

In this review, we have systematically described and analyzed the existing information on possible connections between infection-induced pulmonary events and neurological symptoms. Our analyses might encourage researchers to find early biomarkers and targeted therapeutic approaches to inhibit and/or block the development of neurologic symptoms.

The reported neurological problems in COVID-19 patients might come from the overstimulation of the immune system, and it is presumed that there is a strong involvement of microglia and possibly astroglia in response to peripheral inflammation rather than direct viral infection of the CNS (163). The described events could lead to an interplay between the lung and the brain in the case of SARS-CoV-2 infection, which is based on the fact that the hypoxia and systemic oxidative stress couple the disrupted respiratory epithelium to brain damage. It is known that during COVID-19 infection, there is an increased production of cytokines and chemokines in the periphery, especially in the lung macrophages and in bronchial epithelial cells. When hypoxia occurs owing to difficulties in breathing, a significant amount of proinflammatory factors is released due to the activation of HIF-1α. Certainly, in cases of prolonged and substantial elevation of cytokines in the blood, these can cross the BBB and activate microglia. Similarly, in the case of hypoxia, the lack of oxygen is inevitably sensed by the brain microglia, and this leads to its activation. In a healthy brain, this kind of activation might induce neuroinflammation and potentially lead to neuropsychiatric or neurodegenerative disorders. More severe effects of hypoxia are expected in those patients with pre-existing neurodegenerative diseases, including those which are pre-symptomatic and thus have not yet been diagnosed. In a relevant scenario for Alzheimer’s disease, it has been shown that hypoxia is a major risk factor. Specifically, HIF-1α upregulation in hippocampal microglia leads to reduced Aβ elimination by microglia and hence to increased Aβ-associated neuropathology (164). Knowing that both hypoxia and increased levels of systemic cytokine levels are very likely to cause some degree of damage to the brain, this possibility should not be overlooked and need to be investigated in the future.

Although the predominant clinical symptoms of COVID-19 are pulmonary issues with respiratory manifestations, neurological symptoms are increasingly noted. The loss of smell and taste was reported among the first non-respiratory symptoms, and many people reported otolaryngologic symptoms, headaches, fatigue, and a state called “brain fog” – a condition where patients had trouble thinking clearly (165). All these symptoms suggest possible effects of SARS-CoV-2 on the CNS. Several research groups examined the potential of viral particles of SARS-CoV-2 to invade the CNS tissue, and also the presence of the virus was confirmed in cerebrospinal fluid taken from an encephalitis patient by next-generation sequencing (24) as well as the presence of CSF-specific anti−SARS−CoV−2 antibodies (67). Furthermore, recently in human 3D organoids, it was shown that SARS-CoV-2 is indeed able to infect and kill neurons (166). A preprint publication by Chen et al. (101), claims that human pluripotent stem cell-derived midbrain dopamine neurons are selectively permissive to SARS-CoV-2 infection, with potential long-term implications for the risk of developing Parkinson’s disease-related symptoms. Nevertheless, despite finding further proofs of SARS-CoV-2 protein expression in the brains of the deceased COVID-19 patients, so far, it was not successfully proven that the presence of the virus is directly associated with the neuropathological changes (100). It is important to mention that the study of Song et al. (67) show evidence of the existence of antineuronal autoantibodies that were detectable specifically in COVID-19 patients, while the exact pathogenic relevance of these is yet to be discovered. In the meantime, these autoantibodies could be considered as one of the possible indirect source/contributors to COVID−19 related neuropathology. Presence of such autoantibodies is quite similar to prothrombotic autoantibodies causing occlusion of blood vessels identified recently in Covid-19 patients (75). Moreover, the same study hypothesizes about the similarities between severe COVID−19 phenotypes and those present in patients with lupus and antiphospholipid syndrome, both of which are typical for the presence of anti-phospholipid autoantibodies, and in extremely severe cases, can cause multiple organ failure (75). Additionally, a growing number of case reports describing a connection between SARS−CoV−2 and the Guillain-Barré syndrome (GBS) has emerged recently (167–171). GBS is a state of acute limb paralysis caused typically by an antecedent infectious disease (often a gastrointestinal or respiratory infection) or other immune stimulation that induces an aberrant autoimmune response that targets peripheral nerves and leads to weakness and loss of sensation (172, 173). The reported cases of COVID−19-associated GBS highlight the similarities with cytomegalovirus, Zika virus, and HIV-associated GBS, and thus also suggest a neuroinvasive potential of SARS−CoV−2 (170). At the same time, these findings further underline the importance of immune modulation and autoimmune processes in SARS−CoV−2 infected patients. Therefore, it is very likely that together with other pathological events occurring during COVID−19 illness, detrimental autoimmune effects could contribute to the overall disease progress.

A small Swedish clinical trial demonstrated that CSF of COVID-19 patients with neurological symptoms showed an elevated neurofilament light chain protein (NfL) level that resembles axon injury. Furthermore, the elevated NfL level in the CSF correlated with the seriousness of the neurological symptoms (Glasgow Coma Scale) and was higher in patients between serious and critical disease as compared with mild and moderate disease (174). Based on this, wider clinical trials are much needed to find reliable biomarkers for CNS injury to support the early rehabilitation of COVID-19 patients and to avoid the development of long-COVID. It is currently too early to tell what exactly the long-term effects of COVID-19 on patients’ mental health will be. With so many infected people worldwide, the overall number of patients with neurological complications might grow, and this could induce substantial social and economic costs (175). Moreover, even though the proportion of acute infections with neurological symptoms remains relatively small, neurological complications can lead to lifelong problems. Furthermore, in the cases of patients from the intensive care unit, it had been pointed out that several cases of brain injury could have escaped the attention due to patients being in a state of induced coma, and specific neurological examinations such as imaging were often undertaken only in those people that were slow to wake (83). At the same time, it should be kept in mind that many cases of COVID-related chronic neurological symptoms might not have been reported yet. Considering the global healthcare crisis caused by prioritizing COVID-19 patients with acute and life-threatening conditions, perhaps only after this crisis other COVID-related chronic complications will emerge. Therefore, it is of major importance to provide follow-up studies to determine the long-term neurological effects of this disease. Additionally, it has been observed that the course and the pathological consequences of COVID−19 might differ from patient to patient, future studies on the pathobiological mechanisms and contributing factors will be essential. For example, it would be important to describe the associated risk factors (including those of genetic origin), that can possibly contribute to the different disease outcomes in individual patients. Next, identification of biomarkers that help distinguish between direct viral infection of the brain and immune overactivation would be essential for the selection of proper treatment. Altogether, such knowledge would provide leverage in early recognition and short and long-term management of the disease and perhaps open new opportunities for personalized medicine based on the individual background and risk factors of each patient.

## Author Contributions

LF and AK wrote the original draft. GC contributed to writing of the paper. LF prepared the figures. KF supervised the conceptualization and writing. KG and AD reviewed and edited the final version of the manuscript. All authors contributed to the article and approved the submitted version.

## Funding

This project has received funding from the European Union’s Horizon 2020 Research and Innovation Programme under the Marie Skłodowska-Curie Grant Agreement No. 766124 (PurinesDx) and grant agreements No. 739593 (HCEMM) and No. 814978 (TUBE). Further financial support was granted by the New National Excellence Programme (20391-3/2018/FEKUSTRAT) and the TKP2020 Tématerületi Kiválósági Program (NKFIH-1279-2/2020) by the National Research, Development and Innovation Office, Hungary and the Doctoral Student Scholarship Program of the Co-operative Doctoral Program of the Ministry of Innovation and Technology financed from the National Research, Development and Innovation Fund (Grants No. 994735 and 973877 for AK and GC, respectively).

## Conflict of Interest

LF, AK, GC, AD, and KF are employed by BioTalentum Ltd. The remaining author declares that the research was conducted in the absence of any commercial or financial relationships that could be construed as a potential conflict of interest.

## Publisher’s Note

All claims expressed in this article are solely those of the authors and do not necessarily represent those of their affiliated organizations, or those of the publisher, the editors and the reviewers. Any product that may be evaluated in this article, or claim that may be made by its manufacturer, is not guaranteed or endorsed by the publisher.

## Glossary

**Table:**

ACE2	Angiotensin-converting enzyme 2
ADAM17	A Disintegrin And Metalloprotease 17
Ang II	Angiotensin II
Arg1	Arginase 1
BBB	Blood-brain barrier
BCSFB	Blood-cerebrospinal fluid barrier
BMVEC	Brain microvascular endothelial cells
BRB	Blood-retina barrier
CCL2/3	C–C motif chemokine ligand 2/3
CD	Cluster of differentiation
CD4+/CD8+	Cluster of differentiation 4/8
CNS	Central nervous system
COVID-19	Coronavirus disease 2019
CRP	C-reactive protein
CSF	Cerebrospinal fluid
CSF1R	Colony stimulating factor 1 receptor
CX3CL1	Chemokine (C-X3-C motif) ligand 1
CXCL10	Chemokine (C-X-C motif) ligand 10
DAMP	Damage-associated molecular pattern
DNA	Deoxyribonucleic acid
EGF	Epidermal growth factor
ErbB	Epidermal growth factor receptor family
GBS	Guillain-Barré syndrome
GCSF	Granulocyte colony-stimulating factor
HCoV	Human coronavirus
HIF-1α	Hypoxia-inducible factor 1-alpha
IFIT2	Interferon-induced protein with tetratricopeptide repeats 2
IFN	Interferon
IgG	Immunoglobulin G
IL	Interleukin
iNOS	Inducible nitric oxide synthase
IP-10	Interferon gamma-induced protein 10
iPSC	Induced pluripotent stem cell
IRF3/7	Interferon regulatory factor 3/7
JAK/STAT	Janus kinase/Signal transducer and activator of transcription protein
JMHV	Japanese monkey herpesvirus
LDH	Lactate dehydrogenase
M protein	Myeloma protein
MAPK	Mitogen-activated protein kinase
MAVS	Mitochondrial antiviral signaling
MCP-1	Monocyte chemoattractant protein-1
MDA5	Melanoma differentiation-associated gene 5
MERS-CoV	Middle East respiratory syndrome coronavirus
MHC I/II	Major histocompatibility complex I/II
MHV	Mouse hepatitis virus
MIP1-α	Macrophage Inflammatory Protein 1-α
mTOR	Mechanistic target of rapamycin
mtROS	Mitochondrial reactive oxygen species
N protein	Nucleocapsid protein
NfL	Neurofilament light
NF-κB	Nuclear factor-kappa B
NK cells	Natural killer cells
NLRP3	NLR family pyrin domain containing 3
ORFs	Open reading frames
p40	P40 Protein
PAMP	Pathogen-associated molecular pattern
PDK1	Phosphoinositide-dependent kinase-1
PI3K-Akt	Phosphoinositide 3-kinase - Protein kinase B
PKM2	Pyruvate kinase muscle isozyme M2
PRR	Pattern recognition receptor
RIG-I	Retinoic acid-inducible gene I
RNA	Ribonucleic acid
ROS	Reactive oxygen species
S protein	Spike protein
S100B	S100 calcium-binding protein B
SARS-CoV-1/-2	Severe acute respiratory syndrome coronavirus 1/2
STAT	Signal transducer and activator of transcription protein
TANK	TRAF family member-associated NF-kappa-B activator
TBK1/IKK	TANK-binding kinase 1
TGF-β	Transforming growth factor beta
TLR-3/TLR-7	Toll-like receptor 3/7
TMPRSS2	Transmembrane protease, serine 2
TNF-α	Tumor necrosis factor
TRAF3/6	Tumor necrosis factor receptor-associated factor 3/6
VEGF	Vascular endothelial growth factor
ZO-1	Zonula occludens-1
βCoVs	β-coronaviruses
